# Functional and Structural Connectome Features for Machine Learning Chemo-Brain Prediction in Women Treated for Breast Cancer with Chemotherapy

**DOI:** 10.3390/brainsci10110851

**Published:** 2020-11-12

**Authors:** Vincent Chin-Hung Chen, Tung-Yeh Lin, Dah-Cherng Yeh, Jyh-Wen Chai, Jun-Cheng Weng

**Affiliations:** 1 School of Medicine, Chang Gung University, Taoyuan 33302, Taiwan; cch1966@gmail.com; 2 Department of Psychiatry, Chang Gung Memorial Hospital, Chiayi 61363, Taiwan; 3 Department of Medical Imaging and Radiological Sciences, Chang Gung University, Taoyuan 33302, Taiwan; zlink0587@gmail.com; 4 Breast Medical Center, Cheng Ching Hospital Chung Kang Branch, Taichung 40764, Taiwan; elva3079@yahoo.com.tw; 5 Department of Radiology, Taichung Veterans General Hospital, Taichung 40705, Taiwan; hubt@vghtc.gov.tw; 6 College of Medicine, China Medical University, Taichung 406040, Taiwan; 7 Medical Imaging Research Center, Institute for Radiological Research, Chang Gung University and Chang Gung Memorial Hospital at Linkou, Taoyuan 33302, Taiwan

**Keywords:** breast cancer, chemo brain, machine learning, connectome, resting-state functional magnetic resonance imaging, generalized q-sampling imaging

## Abstract

Breast cancer is the leading cancer among women worldwide, and a high number of breast cancer patients are struggling with psychological and cognitive disorders. In this study, we aim to use machine learning models to discriminate between chemo-brain participants and healthy controls (HCs) using connectomes (connectivity matrices) and topological coefficients. Nineteen female post-chemotherapy breast cancer (BC) survivors and 20 female HCs were recruited for this study. Participants in both groups received resting-state functional magnetic resonance imaging (rs-fMRI) and generalized q-sampling imaging (GQI). Logistic regression (LR), decision tree classifier (CART), and xgboost (XGB) were the models we adopted for classification. In connectome analysis, LR achieved an accuracy of 79.49% with the functional connectomes and an accuracy of 71.05% with the structural connectomes. In the topological coefficient analysis, accuracies of 87.18%, 82.05%, and 83.78% were obtained by the functional global efficiency with CART, the functional global efficiency with XGB, and the structural transitivity with CART, respectively. The areas under the curves (AUCs) were 0.93, 0.94, 0.87, 0.88, and 0.84, respectively. Our study showed the discriminating ability of functional connectomes, structural connectomes, and global efficiency. We hope our findings can contribute to an understanding of the chemo brain and the establishment of a clinical system for tracking chemo brain.

## 1. Introduction

Chemotherapy is a widely used, long-established therapy for cancers; however, it is often associated with long-term toxicity inducing multiple side effects. Cognitive impairment in post-chemotherapy patients has been a serious issue in recent years with the increase in breast cancer survival rate and the number of breast cancer survivors [1,2,3,4]. A term, chemo brain, had been proposed and used to refer to a brain damaged by chemotherapy. Chemotherapy-induced cognitive impairment indicated by impaired cognitive functions such as memory, concentration, multiple operation, executive functions, processing speed, reaction time abilities, and word retrieval, is common among breast, lung, prostate, and ovarian cancer survivors who have received chemotherapeutic agents [3,5,6]. The cognitive impairment in patients can affect work performance and social relationships [6]. A recent study [7], where the daily life of 42 post-chemotherapy participants was evaluated, indicated the requirement of independent diagnostics for cognitive impairment. Although neuropsychological testing is the current standard, neuroimaging has the potential for diagnosing chemo-brain [8]. The National Cancer Institute (NCI) has also called for increased use of neuroscience-based methods. To accomplish this purpose, we conducted a study [9] using several MRI features, including generalized q-sampling imaging (GQI) [10] and resting-state functional magnetic resonance imaging (rs-fMRI) indices, for voxelwise analysis and regional summation analysis to discriminate chemo-brain participants from healthy controls (HCs).

Magnetic resonance imaging (MRI) methods are commonly applied in human brain studies. Blood oxygenation level-dependent functional magnetic resonance imaging (fMRI) has been widely used to explore cerebral function. fMRI is an efficient tool that can demonstrate functional changes in the human brain, and fMRI can be separated into task-based fMRI and resting-state fMRI (rs-fMRI). Compared with task-based fMRI, rs-MRI simplifies the experimental design by instructing the patient to relax and clear the mind and does not require them to follow certain task instructions. Functional imaging can aid in portraying the impacts of breast cancer and treatment.

Diffusion MRI is increasingly applied in the study of many white matter disorders. For example diffusion tensor imaging (DTI) can provide a non-invasive method to investigate brain microstructure and to measure the white matter tract integrity of anatomical connectivity [11,12,13]. However, DTI is unable to detect the crossing or branching patterns of complex regions, and DTI which is based on the Gaussian diffusion and monoexponential b-value dependence reflects the weighted average of all compartments even though the partial volumes of different diffusion compartments may vary [14,15,16]. To better characterize the complicated fiber patterns and distinguish fiber orientations, generalized q-sampling imaging (GQI) have been proposed, providing an opportunity for more accurate, higher-order descriptions when compared to DTI [10]. GQI is a unique q-space reconstruction method derived from q-space imaging. GQI could be applied to a wide range of q-space datasets for a more accurate and sophisticated diffusion MR approach.

Graph theory is a mathematical analysis that uses nodes and edges to represent objects and connections between objects [17]. Thus, researchers can apply graph theoretical analysis for denoting the complex connections of the human brain, which are believed to have segregated as well as integrated functions. In such studies, nodes usually represent brain regions, while edges represent connections between the brain regions. Furthermore, the edges could represent either functional connections (functional connectome; the gray matter network), or structural connections (structural connectome; the white matter network). More specifically, the gray matter network reflects the functional connectivity among brain areas, whereas the white matter network reflects the physical connections of nerve fibers between brain regions. According to previous graph theoretical studies, it is believed that “small-worldness” is a common characteristic of human brains that refers to the high clustering of nodes and short path lengths between nodes that compose an efficient network [18]. The topological coefficients of graph theory, including the mean clustering coefficient (C), normalized clustering coefficient (gamma, γ), local efficiency, characteristic path length (L), normalized characteristic path length (lambda, λ), global efficiency, transitivity, modularity, and the small-worldness index (sigma, σ), can be acquired by calculation.

A previous similar study [19], which conducted a machine learning classification between post-chemotherapy breast cancer survivors (C+), nonchemotherapy-treated breast cancer survivors (C−), and HCs using the connectomes of 19 independent regions of interest (ROIs) and support vector machines (SVMs), achieved an accuracy of 91.23% in C+ vs. C− and an accuracy of 90.74% in C+ vs. HC. fMRI connectivity data were also adopted by researchers who evaluated the accuracy of several models for the prediction of early multiple sclerosis [20], resulting in an accuracy of 85.7% by random forest (RF) and SVM. There was an enormous machine learning study [21] that also used structural connectomes as well as functional connectomes for prediction tasks. In particular, a total of 168 structural connectomes and 1013 functional connectomes were used to predict the cognitive and neuromotor development of preterm neonates and the autism spectrum category of the Autism Brain Imaging Data Exchange (ABIDE) database.

Continuing our previous study [22,23], we further processed our data using graph theoretical analysis and conducted another machine learning classification. Hence, the functional and structural connectomes (connectivity matrices) and their topological coefficients of the participants were used as features in this study. Note that we have two different types of connectomes in the current study, namely, functional connectomes calculated from the rs-fMRI data and structural connectomes calculated from the GQI data.

Logistic regression (LR), decision tree classifier (CART), and xgboost (XGB) were the three best classifiers in our former multiclassifier analysis, which all achieved an accuracy of 84%. Therefore, we prioritized and adopted these models in the current study. In the medical field, various studies have utilized LR and indicated the prominent efficacy of LR, such as predicting clinical drug response [24], differentiating between transition zone cancers and benign prostatic hyperplasia [25], and predicting the risk of a repeat surgical intervention for participants having septic arthritis of the hip [26]. The utilization of CART model has included predicting membrane protein types [27], differentiating brain pilocytic astrocytoma from glioblastoma [28], and diagnosis of biliary atresia in infants with jaundice [29]. The XGB models have won several machine learning competitions, and they have also been applied in many studies, such as coronary plaque characterization [30], predicting glucose metabolism disorder risk [31], and predicting invasive disease-free survival for early-stage breast cancer patients [32]. Theoretically, linear models such as LR were expected to attain the best performance on our high-dimensional dataset compared to ensemble models, RF and XGB. Although SVM, which is compatible with small sample size, should be an appropriate choice, the outcomes of it in our previous studies did not surpass or equal to the best ones; thus, we decided to dispose SVM but focus on the algorithms we used. Among them, LR is the most remarkable algorithm in our point of view. In the aspect of time consumption, LR took the least time to perform predictions, while XGB required the most time to do it. CART and RF are relatively unstable algorithms since their results are sometimes slightly different with the same configuration. Our purpose was to classify the connectomes and topological coefficients of participants into an HC group or a chemo-brain group using the LR, CART, and XGB models.

## 2. Materials and Methods

### 2.1. Participants and Clinical Characteristics

Nineteen post-chemotherapy female BC survivors (age range: 32 to 55 years, mean age: 43.8 ± 6.4 years; years of education: 13.9 ± 2.2; BC stage: I (*n* = 2), II (*n* = 14), III (*n* = 3)) and 20 HCs (age range: 43 to 55 years, mean age: 50.1 ± 2.5 years; years of education: 13.3 ± 2.3) were recruited from Taichung Veterans General Hospital in Taichung, Taiwan (Table 1). All BC survivors had histological confirmation of primary BC and received scans within 6 months after chemotherapy with standard chemotherapeutic agents, i.e., docetaxel and epirubicin. The exclusion criteria for patients with BC were being in a terminal stage of the disease (defined as a life expectancy of less than 1 year), having a treatment history for any cancer other than breast cancer or double cancer, undergoing radiation therapy before the investigation, having an indication of brain metastasis on postcontrast MRI findings or any known brain lesion from previous brain image scans, having any contraindication of MRI scans, and having a history of psychiatric or neurological illnesses or substance-use disorders [22]. The exclusion criteria for healthy controls were a history of psychiatric or neurological illnesses or substance-use disorder, a family history of major psychiatric or neurological illnesses, a current prescription of any psychiatric or psychotropic medications, current pregnancy or breastfeeding, and any contraindication of MRI scans [22]. In addition to chemotherapy, four of the chemo-brain participants received radiation therapy, one received hormone therapy, and nine received surgical therapy. The number of menopausal women in the patients and controls were 5:5. All BC survivors were confirmed with no brain or other metastases screened by brain MRI, chest computed tomography, and bone scan. Regarding menopause status, among all participants, 5 HCs and 5 chemo-brain participants were postmenopausal. All data remained in the functional analysis, however, the data of 1 HC and 1 chemo-brain participant were not included in the structural analysis. As a result, the classification using structural connectomes and topological coefficients contained only 19 HCs and 18 chemo-brain participants, while all 20 HCs and 19 chemo-brain participants were retained in the functional classification.

This study was approved by the Institutional Review Board of Taichung Veterans General Hospital (No. SF14185A). The research assistant explained the research proposal to all participants so that they could understand the research purpose, process, and both their rights and interests, and all participants were compensated when they finished the study. The clinical physicians assessed the physiological status of all participants to ensure they could participate in the MRI examination. All participants participated in the study after providing written informed consent, and all research was performed in accordance with relevant guidelines and regulations. The neuropsychological assessment was performed by the clinical psychologist, and the subsequent MRI examination was performed by the radiologic technologist. The overall process took approximately 1.5 h in the same day. The datasets analyzed during the current study are available from the corresponding author on reasonable request.

We used objective psychological tests and subjective questionnaire to evaluate the cognitive function, mindfulness, and psychological trauma of the participants. The neuropsychological tests included the Mini-Mental State Examination (MMSE), Impact of Event Scale-Revised (IES-R), and Cognitive and Affective Mindfulness Scale-Revised (CAMS-R), and Functional Assessment of Cancer Therapy-Cognitive Function (FACT-Cog). The results of the neuropsychological assessments are shown in Table 1.

### 2.2. MRI Acquisition

A 1.5-T MRI scan (Magnetom Aera, Siemens Medical Systems, Erlangen, Germany) with a standard, 8-channel head coil was used for all the MRI examinations. A total of three examinations were performed for each participant in this study. 1. T1-weighted 3D volume images (magnetization-prepared rapid gradient-echo sequence; TR/TE/TI = 9.11 ms/1.77 ms/450 ms; flip angle = 7°; 124 axial slices; voxel size = 1.0 × 1.0 × 1.4 mm^3^); 2. rs-fMRI (256 volumes; gradient echo-echo planar imaging sequence; 33 axial slices per volume; TR/TE = 2000 ms/30 ms; flip angle = 90°; voxel size = 3.4 × 3.4 × 4.0 mm^3^; temporal resolution = 2 s); 3. GQI (single-shot, diffusion-weighted spin echo-planar imaging sequence; TR/TE = 7200 ms/107 ms; field of view = 256 mm; matrix = 128 × 128; slice thickness = 4 mm; voxel size = 2 × 2 × 4 mm^3^; b-values = 1000, and 2000 s/mm^2^ in 128 noncollinear directions and one null image (b0); number of excitations = 1).

### 2.3. MRI Image Analysis

We used Statistical Parametric Mapping 8 (SPM8, Wellcome Department of Cognitive Neurology, London, UK) based on MATLAB (Math-Work, Natick, MA, USA) for MRI image preprocessing. For the fMRI data, we first conducted slice-timing correction as well as motion correction to reduce the influence caused by slice-timing and motion. Second, we normalized the data to the Montreal Neurological Institute (MNI, Montreal, QC, Canada) standard space [33] and performed spatial smoothing to improve image quality. Finally, linear detrending and bandpass temporal filtering with the frequency range from 0.01 to 0.08 Hz were performed to remove physiological noise by using the functional connectivity toolbox (CONN, The Gabrieli Lab. McGovern Institute for Brain Research MIT, Cambridge, MA, USA). For GQI data, the FSL Eddy correction (FMRIB Software Library, Oxford, UK) was first applied, followed by motion correction, normalizing, spatial smoothing, and normalizing. Eventually, normalized quantitative anisotropy (NQA) was calculated using DSI Studio (National Taiwan University, Taipei, Taiwan) for the subsequent analysis [10].

### 2.4. Graph Theoretical Analysis

For the functional connectome, the functional connectivity toolbox (CONN, The Gabrieli Lab. McGovern Institute for Brain Research MIT, Cambridge, MA, USA) was used, the whole brain was divided into 90 brain regions (45 per hemisphere) with an automated anatomical labeling (AAL) template, each of which was considered a node [34,35]. The brain functional connectivity between two nodes could be represented as an edge. The degree of a node is the number of edges connecting it to the rest of the network, and the degree can be used to characterize the edge distribution of all nodes in the network [36]. The functional MR image was registered to the T1-weighted image and then to the MNI space. The transformation matrix from functional image to MNI space was calculated by the transformation matrices created in the above two register processing steps. We normalized the resting-state functional images to the AAL template in MNI native space spatially, and the functional connectivity matrix was obtained after the functional connectivity analysis. Finally, the graph theoretical analysis was performed by using the functional connectivity matrix.

For the structural connectome, GQI was used to detect the direction of water molecule diffusion in the white matter non-invasively. The pathways of nerve fibers in the brain were reconstructed using fiber assignment by continuous tracking (FACT) with DSI studio. Network edges were established using FACT and the AAL templates, which divided the brain into 90 brain regions in MNI space. The number of virtual fibers connecting each pair of AAL regions was determined, resulting in a 90 × 90 structural connectivity matrix for each participant [17]. The network edges were defined as follows [1]:

(1) $$E=\frac{\mathrm{Fiber count}}{\mathrm{Fiber length}}\times \mathrm{NQA}$$

Finally, both the functional and structural topological properties of the complex network measures across the ranges of network density were obtained by graph theoretical algorithm. The network segregation was evaluated with the mean clustering coefficient (C), normalized clustering coefficient (gamma, γ), and local efficiency, and the network integration was evaluated with the characteristic path length (L), normalized characteristic path length (lambda, λ), and global efficiency. The transitivity, modularity, and small-worldness index (σ) were also evaluated [37]. The density represents the ratio of existing connections to all possible connections from the random network. The differences between groups in network measures below the network density depend on the number of individual networks that fragment in each group, therefore group comparisons below the density are not meaningful [38]. The minimum density value was defined by the limit density of the individual network not to be fragmented, and the maximum density value was defined by the density when the topology indices remained unchanged [39]. The network density range was calculated from 0.16 to 0.45 in 0.01 increments for the functional connectome and from 0.05 to 0.26 in 0.01 increments for the structural connectome.

### 2.5. Machine Learning Algorithms

For machine learning analysis, we used LR, CART, and XGB to classify the connectomes and topological coefficients into a chemo-brain group or an HC group using scikit-learn [40] in python. In addition to the scikit-learn library, we imported the xgboost library for XGB, which is a function called XGBClassifier in the library. Scikit-learn, a convenient, formidable library for machine learning, contains several preprocessing, feature selection methods, algorithms, and so on, which makes the machine learning tasks easier for researchers. LR is a linear classification with a sigmoid activation function, learning the weights for each feature during the training. The essential algorithm of LR is relatively simple and results in the rapid training speed of LR, which is why LR is one of the most fascinating models in terms of high-dimensional and/or sparse data. Nonetheless, LR may acquire inferior performance with low-dimensional data. CART is another prevalent model that sets a series of discriminative questions in each node during the training phase to classify samples into different groups. XGB, one of the most formidable machine learning models, is an ensemble method integrating multiple trees in a single model. Unlike RF, another iconic kind of ensemble method, trees (weak learners) in XGB will attempt to correct the error made by the former tree to build a stronger model. The connectomes and topological coefficients were standardized before being sent into leave-one-out cross-validation (LOOCV). Receiver operating characteristic (ROC) curves and area under the curves (AUCs) were recorded in figures that were generated with the matplotlib library [41].

## 3. Results

### 3.1. Connectome Analysis

**Figure 1:**
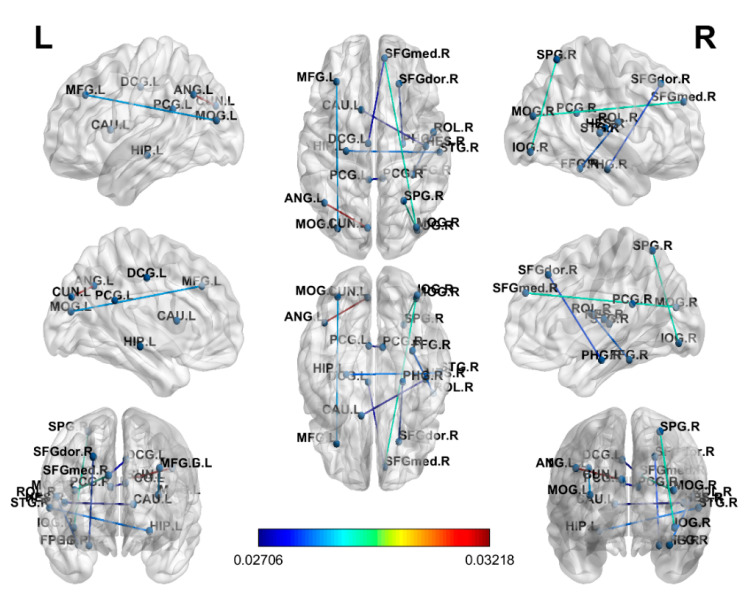
The weighted coefficients of the functional connections in the logistic regression (LR) model. Warmer colors represent higher absolute values of the LR coefficients. A threshold of 0.02706 was set to reduce the connections for visual presentation. Coefficients derived from the attributes of LR, representing the independent coefficient of the features in the decision function. R: right; L: left; M: middle; A: anterior; P: posterior; S: superior; I: inferior; F: frontal; T: temporal P: parietal; O: occipital; G: gyrus.

**Figure 2:**
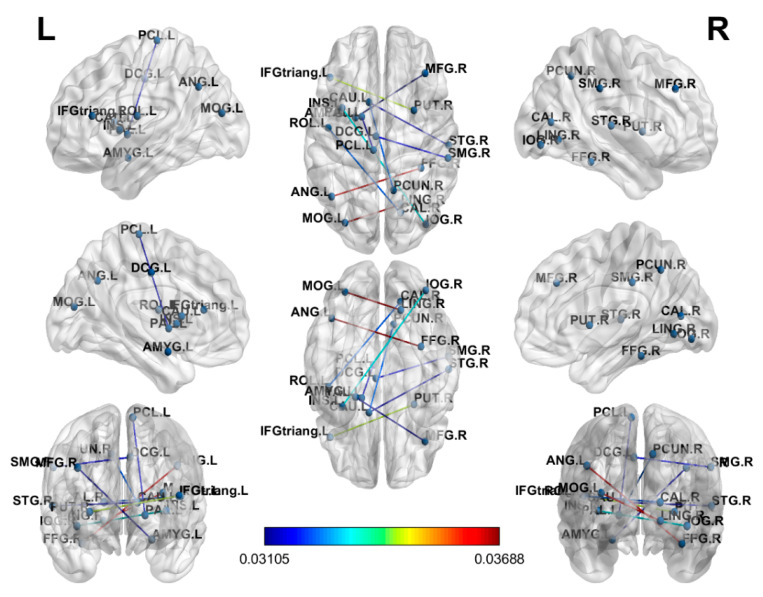
The weighted coefficients of the structural connections in the LR model. Warmer colors represent higher absolute values of the LR coefficients. A threshold of 0.03105 was set to reduce the connections for visual presentation. R: right; L: left; M: middle; A: anterior; P: posterior; S: superior; I: inferior; F: frontal; T: temporal P: parietal; O: occipital; G: gyrus.

In the connectome analysis, LR was the most discriminative model and attained a higher accuracy with functional connectomes (Figure 1) than with structural connectomes (Figure 2). An accuracy of 79.49% with an AUC of 0.93, a sensitivity of 0.80, and a specificity of 0.95 was achieved using functional connectomes, and an accuracy of 71.05% with an AUC of 0.94, a sensitivity of 0.82 and a specificity of 0.91 was achieved using structural connectomes. The figure was drawn using BrainNet [42].

### 3.2. Topological Coefficients Analysis

**Figure 3:**
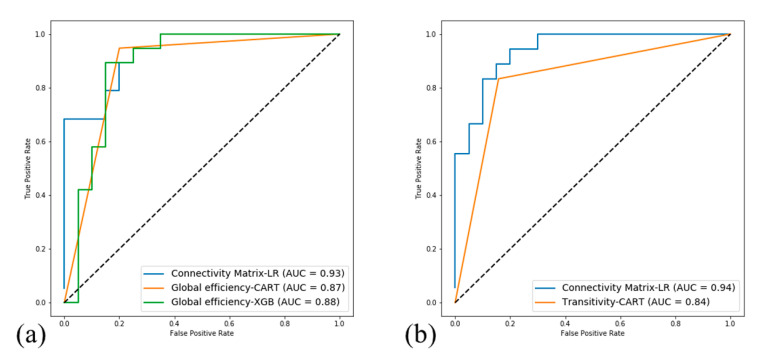
The receiver operating characteristic (ROC) curves and areas under the curves (AUCs) of the predictions. (**a**) Functional connectome analysis and topological coefficient analysis (AUC: connectivity matrix-LR = 0.93; global efficiency-CART = 0.87; global efficiency = 0.88); (**b**) structural connectome analysis and topological coefficient analysis (AUC: connectivity matrix-LR = 0.94; transitivity-CART = 0.84).

In the topological coefficient analysis, machine learning models could not distinguish participants with most features. Apart from most of the topological coefficients, functional global efficiency with CART and XGB and structural transitivity with CART reached accuracies of 87.18%, 82.05%, and 83.78%, with AUCs of 0.87, 0.88, and 0.84, respectively. The ROC curves and AUCs of the connectome analysis and topological analysis are shown in Figure 3.

## 4. Discussion

In the Figure 1, the coefficients derived from the attributes of LR, represent the independent coefficient of the features in the decision function. We found higher absolute values of the functional connection weighted coefficients in the LR model over the frontoparietal and occipital lobes in the cancer patients compared with the healthy controls. The altered brain regions may be associated with the dorsal attention network, and the results were consistent with previous functional neuroimaging studies [43,44]. In Figure 2, higher absolute values of the structural connection weighted coefficients in the LR model over the parietal and occipital lobes were also found in the cancer patients compared with the healthy controls, and the results were consistent with previous structural neuroimaging studies [45].

According to our results, we validated the discriminant ability of functional connectomes in differentiating HCs and chemo-brain participants as a previous study [19] had achieved. Comparing our results with two categories of connectomes, a higher accuracy of 79.49% was obtained with the functional connectomes, and a lower accuracy of 71.05% was obtained with the structural connectomes. However, utilization of structural connectomes could be found in a DL MRI study [46], where researchers used a DL network to predict the survival time of amyotrophic lateral sclerosis patients. They constructed four DL models based on: 1. eight clinical characteristics, 2. structural connectomes from MRI data, 3. cortical thickness and subcortical volume measurements from morphology MRI data, and 4. a combination of the previous three models. A highest accuracy of 84.4% was attained by the fourth model.

For the topological coefficient analysis, the global efficiency of the functional connectome and the transitivity of the structural connectome were the only two topological coefficients that showed an accuracy higher than 80%. The former reached 87.18% and 82.05% with CART and XGB, and the latter reached 83.78% with CART. According to our results, a subtle alteration in global efficiency and transitivity within chemo-brain participants might exist and be detectable with machine learning models. These results might plausibly suggest that there are disturbances in the optimal balance between functional/structural local segregation and global integration in chemo brains. In another study [47] using electroencephalogram (EEG), global efficiency was found to be reduced in young smokers compared with healthy nonsmokers, and the transitivity of major depressive disorder (MDD) participants appeared to decline when compared to HCs [48].

As we mentioned in the Introduction, the choice of machine learning algorithms was based on the outcome of our previous study (6), in which these three algorithms outperformed other algorithms. Additionally, the reason to use standardization remained the same since standardization had increased the classification accuracy. We also attempted to process feature sets to evaluate the performance of the models. For example, we once used a feature set that included all topological coefficients. However, the classification accuracy eventually decreased. From our point of view, this result may be attributed to the fact that only a few topological coefficients vary in chemo-brain participants, and the addition of low discriminative features detrimentally impacted the final performance of the model. Models using combined feature sets, were also considered, e.g., a model using functional and structural connectome. However, in most of the trials, a combined model did not acquire a result surpassing the model using either of them. Finally, we decided to only present results using feature separately.

By advanced computer technology and artificial intelligence, we were able to address numerous problems that may be difficult for humans to perform in an efficient way. For instance, drawing ROIs in images can be time-consuming and tedious, but now we can rely on AI to do the task for us. A recent study [49] provided an overview of the utilization of deep learning (DL)-based segmentation approaches on brain MRI, indicating several models in the past years and the advantages of using automated segmentation methods, such as alleviating intra-expert variabilities and interexpert variabilities. Apart from image segmentation, image reconstruction is another great example of benefits AI has brought to us. Researchers had proposed a DL model [50] that could reconstruct a contrast-enhanced brain MRI image with an image that was enhanced by merely one-tenth of the dosage allowing a reduced gadolinium dose. Using DL to reconstruct fully sampled MR images from undersampled images [29,51] is another popular topic since the undersampling of k-space usually represents a shorter scan time. Furthermore, DL could even generate pseudo-CT scans for attenuation correction in PET/MR imaging [52].

The interpretability of the machine learning model may be limited by the black-box nature of the classifiers. The results from machine learning depend on many a priori decisions regarding the different parameters chosen, and the conclusions may differ somewhat if a different set of parameters were used. It is recommended that future studies should aim to design a new variant to visualize the important brain source patterns and to realize the goal of interpretable machine learning. It is possible that the functional/structural connectome may be affected by behavior, aging, and other clinical issues [53,54], and this could be explored in future studies.

The first limitation was the lack of samples, which has been a common limitation in most MRI studies. However, in the connectome analysis it can be neglected since different values in the connectivity matrix and topological parameters in different density can be treated as one patient (one row in the final feature matrix). To isolate effects of chemotherapy from effects of cancer diagnosis (regardless of type of treatment) [55], we thought that a group of prechemotherapy BC survivors would be most appropriate for comparing pre- and post-chemotherapy participants, which was not achieved in the current study. The control group in the study is older than the patient group. Age may be the confounder and may result in bias close to null. Nonetheless, we can still use machine learning to distinguish patients from control group in the present study but the scale of the findings may underestimate. For our future work, we aim to increase the number of participants, including breast cancer patients and age-matched healthy controls, in our dataset and add prechemotherapy group and post treatment-but no-chemo group to address the disadvantages above. 

## 5. Conclusions

Our study validated the alterations of the connectome in chemo-brain participants and identified the discriminating ability of connectome to machine learning. We are hopeful that this study could help the establishment of a clinically available model to track chemo brain in the future.

## Figures and Tables

**Table 1:** Demographic characteristics and summary of neuropsychological testing and questionnaire evaluation of the all participants in the study.

Characteristic	Breast Cancer Patients after Chemotherapy (*n* = 19)				Healthy Controls (*n* = 20)					
	Mean	SD	Min	Max	Mean	SD	Min	Max	*p*-Value	Effect Sizes
Age (years)	44.84	6.56	32	55	49.95	2.61	43	55	0.004	−1.03
Education (years)	14.00	2.61	9	16	13.2	2.26	9	16	0.274	0.33
MMSE	28.11	1.29	26	30	28.35	1.46	25	30	0.583	−0.17
CAMS-R	33.63	4.91	24	42	33.85	3.94	27	40	0.879	−0.05
IES-R	14.26	23.91	0	64	8.60	12.57	0	38	0.366	0.30
**FACT-Cog**										
Perceived cognitive impairments	53.26	10.49	34	67	59.45	6.88	47	70	0.035	−0.70
Comments from others	14.00	2.47	7	16	14.30	1.84	11	16	0.669	−0.14
Perceived cognitive abilities	17.21	5.78	2	26	18.00	5.49	0	24	0.664	−0.14
Impact on quality of life	11.63	3.52	4	16	14.10	2.05	10	16	0.010	−0.86
Total score	102.37	19.24	53	131	112.50	12.31	90	134	0.056	−0.63
**Breast cancer stage**	***n***	**%**			***n***	**%**				
0	0	0			N/A	N/A				
I	2	10.53			N/A	N/A				
II	14	73.68			N/A	N/A				
III	3	15.79			N/A	N/A				
IV	0	0			N/A	N/A				
Chemotherapeutic drugs (Taxotere and Epirubicin)	19	100.00			N/A	N/A				
Radiation therapy	4	21.05			N/A	N/A				
Hormonal treatment	1	5.26			N/A	N/A				
Menopausal	5	26.32			5	25.00				

Abbreviations: MMSE: Mini-Mental State Examination; CAMS-R: Cognitive and Affective Mindfulness Scale-Revised; IES-R: Impact of Event Scale—Revised; FACT-Cog: Functional Assessment of Cancer Therapy-Cognitive Function; SD: standard deviation; N/A: not applicable.

## Abbreviations

AAL	automated anatomical labeling
ABIDE	Autism Brain Imaging Data Exchange
AUC	areas under curve
BC	breast cancer
C	mean clustering coefficient
C+	breast cancer survivors
C−	nonchemotherapy-treated breast cancer survivors
CAMS-R	Cognitive and Affective Mindfulness Scale-Revised
CART	decision tree classifier
DL	deep learning
EEG	electroencephalogram
FACT	fiber assignment by continuous tracking
FACT-Cog	Functional Assessment of Cancer Therapy-Cognitive Function
GQI	generalized q-sampling imaging
HC	healthy control
IES-R	Impact of Event Scale–Revised
L	characteristic path length
LOOCV	leave-one-out cross-validation
LR	logistic regression
MDD	major depressive disorder
MMSE	Mini-Mental State Examination
MNI	Montreal Neurological Institute
NQA	normalized quantitative anisotropy
RF	random forest
ROI	region of interest
rs-fMRI	resting-state functional magnetic resonance imaging
SPM	Statistical Parametric Mapping
SVM	support vector machine
XGB	xgboost
γ	normalized clustering coefficient
λ	normalized characteristic path length
σ	small-worldness index
